# Allergen hybrids – next generation vaccines for Fagales pollen immunotherapy

**DOI:** 10.1111/cea.12250

**Published:** 2014-02-22

**Authors:** U Pichler, M Hauser, H Hofer, M Himly, E Hoflehner, M Steiner, S Mutschlechner, K Hufnagl, C Ebner, A Mari, P Briza, B Bohle, U Wiedermann, F Ferreira, M Wallner

**Affiliations:** 1 Christian Doppler Laboratory for Allergy Diagnosis and Therapy, University of SalzburgSalzburg, Austria; 2 Institute of Specific Prophylaxis and Tropical Medicine, Medical University of ViennaVienna, Austria; 3 Christian Doppler Laboratory for Immunomodulation, Department of Pathophysiology and Allergy Research, Medical University of ViennaVienna, Austria; 4 Allergieambulatorium ReumannplatzVienna, Austria; 5 Center for Molecular Allergology, IDI-IRCCSRome, Italy; 6 Allergome-Allergy Data Laboratories s.c.Latina, Italy

**Keywords:** allergen-specific immunotherapy, birch pollen allergy, Fagales pollen allergy, hybrid protein, immunomodulation, protein remodelling

## Abstract

**Background:**

Trees belonging to the order of Fagales show a distinct geographical distribution. While alder and birch are endemic in the temperate zones of the Northern Hemisphere, hazel, hornbeam and oak prefer a warmer climate. However, specific immunotherapy of Fagales pollen-allergic patients is mainly performed using birch pollen extracts, thus limiting the success of this intervention in birch-free areas.

**Objectives:**

T cells are considered key players in the modification of an allergic immune response during specific immunotherapy (SIT), therefore we thought to combine linear T cell epitope-containing stretches of the five most important Fagales allergens from birch, hazel, alder, oak and hornbeam resulting in a Fagales pollen hybrid (FPH) molecule applicable for SIT.

**Methods:**

A Fagales pollen hybrid was generated by PCR-based recombination of low IgE-binding allergen epitopes. Moreover, a structural-variant FPH4 was calculated by *in silico* mutagenesis, rendering the protein unable to adopt the Bet v 1-like fold. Both molecules were produced in *Escherichia coli*, characterized physico-chemically as well as immunologically, and tested in mouse models of allergic sensitization as well as allergy prophylaxis.

**Results:**

Using spectroscopic analyses, both proteins were monomeric, and the secondary structure elements of FPH resemble the ones typical for Bet v 1-like proteins, whereas FPH4 showed increased amounts of unordered structure. Both molecules displayed reduced binding capacities of Bet v 1-specific IgE antibodies. However, in a mouse model, the proteins were able to induce high IgG titres cross-reactive with all parental allergens. Moreover, prophylactic treatment with the hybrid proteins prevented pollen extract-induced allergic lung inflammation *in vivo*.

**Conclusion:**

The hybrid molecules showed a more efficient uptake and processing by dendritic cells resulting in a modified T cell response. The proteins had a lower IgE-binding capacity compared with the parental allergens, thus the high safety profile and increased efficacy emphasize clinical application for the treatment of Fagales multi-sensitization.

## Introduction

Allergy vaccine development is a time- and cost-demanding process, especially when reaching the phase of clinical trials. Thus, it is essential to carefully select the most promising concepts and candidate molecules early in pre-clinical stages and to invest time and research in the optimization process of potential vaccine candidates. Allergen-specific immunotherapy (SIT) is probably the only allergy treatment capable of altering the course of the disease 1. The repeated application of the disease electing allergens initiates desensitization of the allergic patient. Allergen extracts represent the active ingredient of SIT products; however, several problems are associated with extract immunotherapy. The difficult to standardize and sometimes poorly defined extracts bear the risk of treatment-induced adverse side-effects 2. Moreover, SIT with allergen extracts can be ineffective, thus limiting treatment efficacy. Successful attempts to replace allergen extracts with recombinant allergens have been reported 3,4. There are few allergies, one of them being birch pollen allergy, where a single major allergen dominates the disease, thus a vaccine product based on a single allergen or derivative thereof would be sufficient for effective SIT treatment. Still birch pollen allergy usually does not come alone. Botanically, birch trees belong to the order of Fagales, which represent the major cause of spring pollinosis in the temperate climate zones of the Northern Hemisphere. Fagales trees show a distinct geographical distribution where birch and alder are endemic in the northern parts of Europe and North America, while hazel, hornbeam and oak prefer a warmer climate, thus populating rather the southern parts of these continents. Co-populations of all five species are frequently found in temperate climate zones 5. Several Betulaceae trees including alder, hazel and hornbeam have the potential to initiate sensitization to Bet v 1-like allergens in susceptible individuals resulting in the production of highly cross-reactive IgE antibodies 6. Therefore, SIT of Fagales allergic patients is often performed using a mixture of several tree pollen extracts, usually birch, hazel, and alder 7. As T cells are considered key players in the modification of an allergic immune response during SIT, this approach has the potential to modulate the T cell response towards multiple tree pollen allergens 1, thus increasing treatment efficacy. Applying this concept, we thought to combine linear T cell epitope-containing stretches of the five most important Fagales allergens (Bet v 1, Aln g 1, Cor a 1, Car b 1, and Que a 1) to a single full-length hybrid molecule termed Fagales pollen hybrid (FPH). The protein has the same overall length and a similar structure as its parental allergens. By using naturally occurring low IgE-binding isoforms of each allergen, antibody-mediated side-effects should be reduced. In addition, a second hybrid (FPH4) was designed as a fold variant of FPH by adopting the design concept of the low IgE binding highly immunogenic Bet v 1 derivative BM4 8. Both molecules were produced in *Escherichia coli*, extensively characterized physico-chemically as well as immunologically and tested in mouse models of allergic sensitization as well as allergy prevention.

## Material and methods

### Patients and sera

Sera of Fagales pollen-allergic patients showing a typical case history, positive *in vivo* skin prick test, and *in vitro* IgE to birch pollen and hazel pollen extracts (Thermo Fisher, Uppsala, Sweden) were collected for the study (Table S1). Informed written consent was obtained from all donors. Experiments including human blood samples were approved by the Ethic Committee of the Medical University and General Hospital of Vienna (no. EK028/2006) and the Institutional Review Board at IDI-IRCCS (n.106-CE-2005).

### Recombinant allergens

Recombinant reference allergens (Bet v 1.0101, Bet v 1.0102, Aln g 1.0101, Cor a 1.0104, Car b 1.0109 and Que a 1.0301) were produced according to published methods 9,10 and characterized physico-chemically 6. Endotoxin content was < 0.3 ng/mg for Bet v 1.0101, < 0.3 ng/mL for Bet v 1.0102, < 0.3 ng/mL for Aln g 1.0101, 0.8 ng/mL for Cor a 1.0104, < 0.3 ng/mg for Car b 1.0109, and < 0.3 ng/mg for Que a 1.0301 as determined by limulus amoebocyte lysate (LAL) assay (Associates of Cape Cod, Inc., East Falmouth, MA, USA).

### Cloning of FPH and FPH4

**Figure 1:**
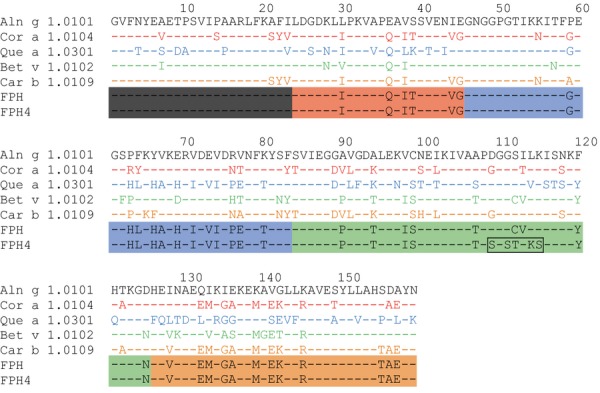
Multiple sequence alignment of amino acid sequences deduced from *bet v 1.0102* (X77266) (green), a*ln g 1.0101* (S50892) (black), *cor a 1.0104* (X70998) (red), c*ar b 1.0109* (EU283857) (orange), and *que a 1.0301* (EU283863) (blue). The amino acid sequences stretches assembling the Fagales pollen hybrids (FPHs) and FPH4, respectively, are coloured according to colours of the parental allergens. The exchanged sequence of FPH4 is boxed.

To construct the hybrid molecule FPH (Fig. 1), PCR amplified fragments of *bet v 1.0102* (X77266), *aln g 1.0101* (S50892), *cor a 1.0104* (X70998) *car b 1.0109* (EU283857), and *que a 1.0301* (EU283863) were generated using primers Aln_F (5′AGGGCGCCATGGGTGTTTTCAATT3′), Aln_R (5′GAGCTTATCGCCATCAAGG3′), Cor_F (5′ CCTTGATGGCGATAAGCTC3′), Cor_ R (5′ GTTCCAGGTCCTCCATTTCCTCCAACGTTTTCAACGCTGGTAATAGC3′),Que_ F (5′CAGCGTTGAAAACGTTGGAGGAAATGGAGGACCTGGAAC3′), Que_ R (5′CCGCCCTCAATCACGCTAAAGCTATATGTAAAGTTTTC3′), Bet_ F (5′GAAAACTTTACATATAGCTTTAGCGTGATTGAGGGCGG3′), Bet_ R (5′CTGCGTTAACCTCATGGTTGCCTTTGGTGTG3′), Car_F (5′CACACCAAAGGCAACCATGAGGTTAACGCAG3′), Car_R (5′CGCGAATTCTTAGTTGTATTCAGCAGTGTGTGCC3′), gel-purified (Wizard® SV Gel and PCR Clean up system, Promega, Madison, WI, USA), recombined in a primerless PCR via homologous DNA stretches, and assembled to full-length genes. The newly assembled genes were PCR amplified with the primer pair Aln_F and Car_R and cloned into a pET 28b expression vector. FPH4 was generated by site-directed mutagenesis of FPH using the primers FPH4_F (5′GCAACCCCTagTGGAaGcacCaTCaaGAgtATCAGCAAC3′) and FPH4_R (5′GTTGCTGATacTCttGAtGgtgCtTCCActAGGGGTTGC 3′) as previously described 8.

### Recombinant production of FPH and FPH4

Expression plasmids were transformed into *E. coli* BL21 Star™ (DE3) (Life Technologies, Grand Island, NY, USA), cells were grown to OD600 of 0.8 in LB medium supplemented with 25 mg/L kanamycin, protein expression was induced by addition of 0.5 mm isopropyl-β-D-thiogalactopyranoside (IPTG), and cells were grown for 20 h at 16°C and harvested by low speed centrifugation. Cells were resuspended in 25 mm sodium phosphate buffer pH 8, lysed and supplemented with 0.5 m sodium chloride, and 150 mm NaH_2_PO_4_. FPH was purified using a phenyl-Sepharose column followed by a polishing step with a Q-Sepharose column (GE Healthcare, Little Chalfont, UK). Expression of FPH4 was performed as described for FPH. After harvesting, the cell pellet was resuspended in PBS 50 mm Tris Base, 1 mm EDTA, 0.1% Triton X-100, the cells were lysed, proteins were precipitated by centrifugation, and thereafter, the pellet was washed with 50 mm Tris Base, 1 mm EDTA, 1% Triton X-100, followed by a wash step with 25% EtOH, 5 mm sodium phosphate pH 7.4. Insoluble proteins were precipitated, dissolved in 6 m Urea, 20 mm sodium phosphate pH 7.4, and the solution was passed over a Q-sepharose column (GE Healthcare). The flow through containing FPH4 was purified using a phenyl-Sepharose column, followed by a polishing step via size exclusion chromatography (GE Healthcare). After dialysis against 10 mm sodium phosphate buffer, pH 7.4, recombinant proteins were stored at −20°C. Endotoxin content of < 0.3 ng/mg for FPH and < 0.3 ng/mg protein for FPH4 was measured by LAL assay (Associates of Cape Cod, Inc).

### Physicochemical analysis of recombinant proteins

Protein purity was analysed by SDS-PAGE, identity by amino acid analysis and mass spectrometry, secondary structure by circular dichroism spectroscopy in 5 mm sodium phosphate buffer pH 7.4 at 20°C, the hydrodynamic radius in solution by dynamic light scattering (DLS) in 5 mm sodium phosphate buffer pH 7.4 at 20°C, and aggregation behaviour by online high-performance size exclusion chromatography (HP-SEC) light scattering in PBS pH 7.4 at 20°C 8,11.

### ELISA experiments

Human sera were diluted 1/10, added to pre-coated (100 ng antigen/well), blocked Maxisorp plates (Nalge Nunc, Rochester, NY, USA), and incubated overnight at 4°C. Bound IgE was detected with alkaline phosphatase-conjugated monoclonal anti-human IgE antibodies (BD Biosciences, San Jose, CA, USA) using a chromogenic substrate 8. Measurements were performed as triplicates; results are presented as mean OD values. For inhibition ELISA, plates were coated with 2 μg/mL antigen overnight at 4°C. Sera diluted 1/10 were pre-incubated with titrated antigens overnight at 4°C in a final volume of 75 μL and thereafter transferred to coated and blocked ELISA plates. IgE detection was performed as described for ELISA. For detection of murine IgG, animal sera were titrated starting at a dilution of 1/10 in dilution steps of 1/10 and added to antigen coated plates. After washing, bound IgG antibodies were detected with either alkaline phosphatase-conjugated monoclonal rat anti-mouse IgG1 or rat anti-mouse IgG2a antibodies (Southern Biotech, Birmingham, AL, USA) followed by chromogenic substrate development. Measurements were performed as triplicates; results are presented as end-point titres. Murine IgE was detected at a serum dilution of 1/10 using a purified rat anti-mouse IgE antibody (clone R35-72, BD Biosciences). Measurements were performed as duplicates.

### Animal immunization experiments

Female BALB/c mice (Charles River Laboratories, Wilmington, MA, USA) were purchased at 8–10 weeks of age and used for experiments 4 days after arrival. Animals were injected subcutaneously (s.c.) with 5 μg antigen adsorbed to Alugel-S (Serva, Heidelberg, Germany) given as two 50 μL s.c. injections administered bilaterally in the lumbar region and boosted on day 14, 21, and 42. Sera were collected on day 0, 21, and 49. Animals were killed on day 50 for analysis of splenocytes. Per group 5 animals were tested. Serum IgG1 and IgG2a were analysed by ELISA, and murine IgE response was analysed by mediator release assays as previously described 12. In brief, RBL-2H3 was passively sensitized with diluted murine serum IgE, and mediator release was triggered by the addition of antigen at 0.3 μg/mL and is expressed as percentage of total enzyme content of Triton X100-treated cells 12. ELISpot assays were performed to determine antigen-specific IL-4 or IFN-γ-secreting splenic lymphocytes using matched pair mAbs for IL-4 or IFN-γ detection, respectively 8. All animal experiments were conducted according to National guidelines approved by the Austrian Ministry of Science (BMWF-66.012/0011-II/10b/2010).

### Proliferation assay

After immunization with 5 μg/mouse of an equimolar antigen mix consisting of Bet v 1.0101, Aln g 1.0101, Cor a 1.0104, Car b 1.0109, and Que a 1.0301, female BALB/c mice (age 8–10 weeks, Charles River Laboratories) received three booster immunizations s.c. in weekly interval and were killed at day 7 after the last boost. CD4^+^ splenic T cells were isolated using appropriate MACS bead technology (CD4^+^ T Cell Isolation Kit; Miltenyi Biotec, Auburn, CA) and subsequently stained with CFSE (Life Technologies). Cells were co-cultured with 1 × 10^5^/well antigen-pulsed (3 μg/mL) BMDCs in a 96-well plate in RPMI medium supplemented with 10% fetal bovine serum (FCS), 2 mm L-glutamine, 100 U/mL penicillin, and 0.1 mg/mL streptomycin (PAA Laboratories GmbH, Pasching, Austria). At day 4, cells were stained with PerCP/Cy5.5-conjugated anti-mouse CD4^+^ antibodies (eBioscience, San Diego, CA, USA), and proliferative responses were visualized by a decrease of CFSE-stained cells. Flow cytometric assays were performed on a FACS Canto II (BD Biosciences) and analysed using DIVA software (BD Biosciences). For cytokine analyses of splenocyte supernatants 2 × 10^5^ cells/well were re-stimulated with 20 μg of the respective antigen and after 72 h incubation at 37°C, 7% CO_2_ supernatants were analysed using a mouse TH1/TH2/TH17/TH22 13plex FlowCytomix kit (eBioscience). Data analysis was performed using the FlowCytomix Pro Software (eBioscience).

### Prophylaxis model of Fagales allergy

BALB/c mice (*n* = 7 per group; age 8–10 weeks Charles River Laboratories) were pre-treated with either (i) Bet v 1.0101, (ii) an equimolar mix of Bet v 1.0101, Aln g 1.0101, Cor a 1.0104, Car b 1.0109, and Que a 1.0301, (iii) FPH, (iv) FPH4, or (v) PBS pH 7.4 through three intranasal administrations of 10 μg antigen in total volume of 20 μL PBS in weekly intervals (day 0, 7, 14). At days 21, 35, 49, and 63, animals were immunized subcutaneously (s.c.) with 5 μg antigen mix (equimolar mix of Bet v 1.0101, Aln g 1.0101, Cor a 1.0104, Car b 1.0109, and Que a 1.0301) adsorbed to Alugel-S (Serva) given as two 50 μL s.c. injections administered bilaterally in the lumbar region. At day 70 and 73, animals received an aerosol challenge with 10 mL of a mix of tree pollen extracts from birch, alder, hazel, hornbeam, and oak, in a 1 mg/mL solution. Equal amounts of total protein determined by Bradford assay were used for preparing the extract mix. Blood samples were drawn on days −1, 67, and 75; animals were killed on day 76. Broncho-alveolar lavage (BAL) fluids in PBS pH 7.4 were added in triplicates to antibody coated plates for IL-5 detection. ELISAs were performed using a mouse-specific IL-5 ELISA kit (eBioscience) followed by chromogenic detection. Eosinophilia in BAL fluids was determined by flow cytometry with negative gating, using anti-mouse Gr-1 PE-conjugated antibody clone RB6-8C5 and anti-mouse CD45 FITC-conjugated antibody clone IBL-5/25 (Immunotools, Friesoythe, Germany). RBL assays were performed as described using serial dilutions of an equimolar allergen mix.

### In vitro antigen uptake

Bone marrow-derived dendritic cells (BMDCs) from BALB/c mice were harvested from culture at day 7, and 2 × 10^5^ BMDCs were loaded with 1 μg antigen. Cells were incubated in RPMI supplemented with 10% FCS, 2 mm L-glutamine, 100 U/mL penicillin and 0.1 mg/mL streptomycin, (PAA Laboratories GmbH), and 10 ng/mL GM-CSF (PeproTech GmbH, Hamburg, Germany) for the indicated time points at 37°C prior to labelling with specific antibodies against CD86 and CD11c (all from eBioscience). Flow cytometric assays were performed on a FACS Canto II (BD Biosciences) and analysed using DIVA software (BD Biosciences).

### Statistical analysis

Statistical evaluations of ELISA and inhibition ELISA with human sera were performed using paired-samples *t*-tests, mediator release assays with human IgE were analysed with Wilcoxon rank-sum test, and student’s *t*-tests were used to calculate statistics of experiments with murine antibodies, cells, or cytokines, respectively. A value of *P *<* *.05 was considered statistically significant (*P < 0.05; **P < 0.01, ***P < 0.001).

## Results

### Construction of Fagales pollen hybrid molecules

Two Fagales pollen hybrid molecules were designed based on epitope recombination of five different allergenic Bet v 1 homologues from birch, alder, hazel, hornbeam, and oak (Fig. 1). The epitope selection for reassembling a full-length hybrid molecule was based on previously identified T cell reactive linear epitopes of the respective allergens 13–16. To reduce potential IgE-binding properties of hybrid proteins in the forefront, only allergen isoforms with reported low IgE-binding capacity were considered for epitope grafting 16,17. Linear epitopes were recombined using conserved regions of high amino acid as well as nucleotide sequence homologies for the cross-over events. Two variants of a hybrid molecule were designed: first, the variant FPH harbouring secondary structure elements comparable to those found in parental allergens and second, a fold variant thereof termed FPH4. To genetically modify the Bet v 1-like fold of FPH, an epitope previously identified as critical for Bet v 1 to adopt its native structure was replaced by the corresponding region of apple Mal d 1, resulting in the generation of FPH4 8.

### FPH displays Bet v 1-like secondary structure elements, whereas FPH4 has a modified fold

**Figure 2:**
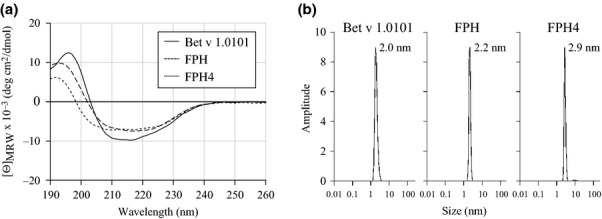
(a) Circular dichroism spectra of Bet v 1.0101, Fagales pollen hybrid (FPH), and FPH4 were recorded at 20°C. All spectra are baseline corrected and presented as mean residue molar ellipticity. (b) The hydrodynamic radius of proteins was determined by dynamic light scattering in sodium phosphate buffer at 20°C.

Secondary structure elements of FPH and FPH4, respectively, were determined by circular dichroism spectroscopy. Fagales pollen hybrid displays secondary structure elements typical for Bet v 1-like allergens, whereas the secondary structure of FPH4 shows pronounced levels of unordered structural elements, indicated by a clear left shift of the curve intersection with the *X*-axis (Fig. 2a). To determine the stability of the protein preparations, several repeated freeze and thaw cycles (> 3) were performed and the CD spectra were compared to untreated protein. No changes in secondary structure elements were detected (data not shown). The hydrodynamic radius of the hybrid proteins determined by DLS was 2.2 nm for FPH and 2.9 nm for FPH4, respectively (Fig. 2b). WT Bet v 1 homologues used as parental allergens have hydrodynamic radii between 2.0 and 2.4 nm, whereas the hydrodynamic radius of the Bet v 1 fold variant BM4 used as design template of FPH4 was determined to be 3.0 nm 6,8. Thus, the DLS data provide strong evidence that the overall shape of FPH resembles the shape of WT Bet v 1-like allergens, whereas the FPH4 shape shows distinct structural deviations. Investigation of the aggregation behaviour of FPH and FPH4 in PBS at 20°C by DLS revealed that both proteins were monomeric to at least 95% at these measurement conditions.

### IgE binding is strongly reduced in hybrid proteins

**Figure 3:**
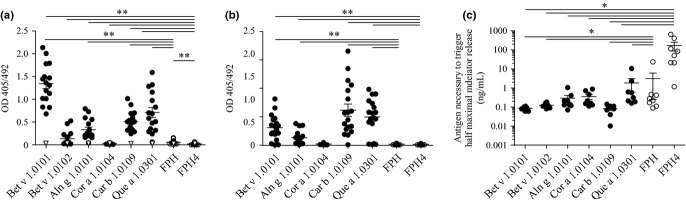
(a) For Austrian (*n* = 16) as well as (b), Italian (*n* = 18) patients′ serum IgE binding (filled circles) was analysed by ELISA, as control normal human serum was used (open triangles). (b) Allergenic activity of candidate molecules was tested in mediator release assays using Fagales allergic patients′ sera (*n* = 8). *P < 0.05, **P < 0.01.

In ELISA experiments using either sera from Austria or Italy, FPH shows significantly reduced human serum IgE binding compared with parental allergens except for the per se low IgE-binding isoforms Bet v 1.0102 or Cor a 1.0104, respectively. For Austrian patients, the fold variant FPH4 exhibited significantly reduced IgE binding compared with all WT allergens and even compared with the hybrid FPH (Fig 3a), whereas for Italian, patients IgE binding was significantly reduced for wild-type allergens except Cor a 1.0104. Moreover, we did not observe a significant difference between FPH and FPH4 (Fig. 3b). The reduced IgE binding of both hybrids translated into decreased receptor cross-linking on passively sensitized RBL cells. Thus, compared with Bet v 1.0101, 37-times more FPH (*P *<* *0.05) and 1988-times more FPH4 (*P *<* *0.05) were necessary to trigger 50% release of inflammatory mediators (Fig. 3c). Of note, significantly more FPH4 was necessary to induce half-maximal mediator release than any of the tested Fagales allergens, which emphasizes the low IgE-binding activity of the molecule. In inhibition assays using Austrian sera (Figure S1), we found that Bet v 1.0101 and Aln g 1.0101 inhibited IgE binding to themselves to around 95% even at a concentration of 0.1 mg/mL, and the inhibition rate for Car b 1.0109 at that concentration was approximately 85%. However, the inhibition of FPH to the respective parental allergens was at 0, 13, and 0%, respectively, and for FPH4, the inhibition rates were 3, 21, and 4%. Only inhibition rates of both hybrids to Cor a 1.0104 and Que a 1.0301 were higher, which might be explained with the generally low reactivity of both parental allergens in ELISA. For Cor a 1.0104, the inhibition with itself at 0.1 mg/mL was 57% compared with 35 and 43% with FPH and FPH4x, respectively. For Que a 1.0301, the inhibition was 41% compared with 6 and 11% obtained with the hybrid proteins.

### Fagales hybrids induce high levels of cross-reactive antibodies

**Figure 4:**
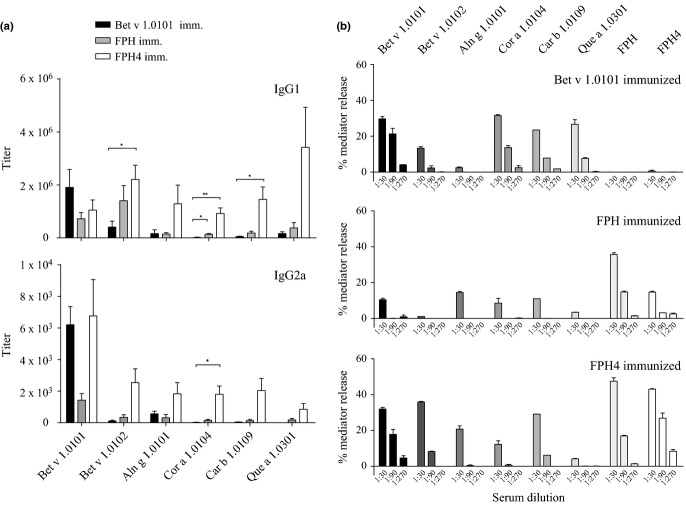
(a) Mice (*n* = 5 per group) were immunized four times with either Bet v 1.0101 (black bars), Fagales pollen hybrid (FPH) (grey bars), or FPH4 (white bars) and specific IgG1 as well as IgG2a levels were determined by ELISA. (b) Functional IgE was analysed by mediator release assays of pooled immune sera at different dilutions upon stimulation with WT allergens or hybrid proteins, respectively. *P < 0.05, **P < 0.01.

The induction of blocking IgG antibodies cross-reactive with WT Fagales allergens is crucial for a possible therapeutic application of hybrid molecules as vaccines; thus, mice were immunized with either antigen (FPH or FPH4), and antibody cross-reactivity was tested by analysing IgG1 as well as IgG2a titres (Fig. 4a). As control, mice were immunized with Bet v 1.0101 plus each of the parental allergens (data not shown). Both hybrids induced a strong cross-reactive IgG response, which was significantly stronger for FPH4 compared with FPH. Bet v 1.0101 elicited a strong IgG response only against itself, and cross-reactive IgG antibodies were significantly lower compared with FPH4. A similar picture was observed for the other Fagales pollen allergens (data not shown). Bet v 1.0101, FPH, and FPH4 all induced cross-reactive IgE antibodies, which was particularly interesting because Bet v 1.0101 failed to elicit a cross-reactive IgG response (Fig. 4b). Despite the fact that the IgE antibodies induced by Bet v 1 cross-reacted with the other wild-type allergens, no IgE cross-reactivity was found with either of the hybrids, reflecting the picture observed with human IgE.

### Hybrid proteins can deviate the TH response in a mouse immunization model

**Figure 5:**
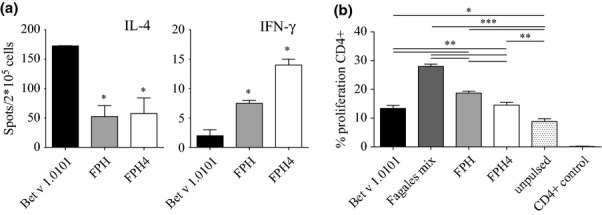
(a) Splenocytes from immunized mice were stimulated with immunogens, and ELISpot values are presented as mean cytokine-secreting cells per 10^5^ cells ± SEM (b). Proliferative response of pooled CFSE-labelled splenic CD4^+^ cells from multi-sensitized animals (*n* = 15) were stimulation with the respective antigens. *P < 0.05, **P < 0.01, ***P < 0.001.

Splenocytes isolated from immunized mice were stimulated with the respective immunogens, and then, IL-4 and IFN-γ secreting T cell were analysed by ELISpot assays (Fig. 5a). Both hybrids induced significantly lower levels of IL-4 producing T cells but significantly elevated levels of IFN-γ secreting cells compared with Bet v 1.0101. Moreover, CD4^+^ T cells from multi-sensitized mice immunized with equimolar mixtures of five different recombinant Bet v 1-homologues from birch, alder, hazel, hornbeam, and oak were stimulated with *in vitro* pulsed murine bone marrow-derived dendritic cells (BMDC), and proliferative responses were analysed (Fig. 5b). Both hybrids showed significantly increased T cell activating properties compared with Bet v 1.0101 alone, Bet v 1.0101 in combination with the other four Bet v 1-homologues resulted in highest T cell proliferation.

### Prophylactic allergy treatment with hybrid molecules can ameliorate allergic symptoms

**Figure 6:**
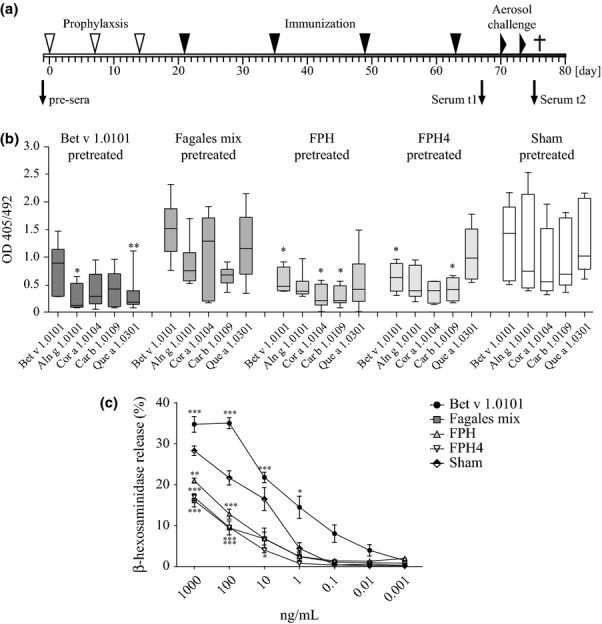
(a) Schematic representation of the allergy prophylaxis model. For prophylactic treatment, animals either received Bet v 1.0101; an equimolar mix of Bet v 1.0101, Aln g 1.0101, Cor a 1.0104, Car b 1.0109, and Que a 1.0301; Fagales pollen hybrid (FPH); FPH4; or sham. For immunization, an equimolar mix of Bet v 1.0101, Aln g 1.0101, Cor a 1.0104, Car b 1.0109, and Que a 1.0301 was used. The aerosol challenge was performed with a mix of birch, alder, hazel, hornbeam, and oak pollen extracts. (b) IgE response of mice (*n* = 7 per group) at t1 after prophylactic treatment with either Bet v 1.0101, an allergen mix, FPH, or FPH4 followed by multi-sensitization with a mix consisting of five Fagales extracts was determined by ELISA. Recombinant WT allergens were immobilized on the solid phase. (c) Mediator release assays were performed with murine sera (t1). Means ± SEM are indicated.

**Figure 7:**
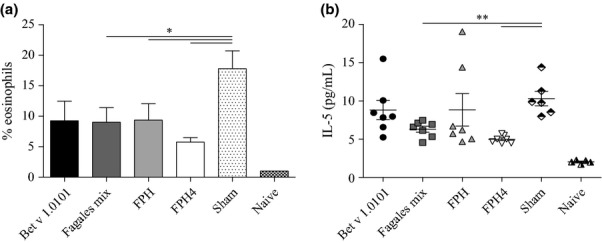
(a) Eosinophil counts and (b). IL-5 of Broncho-alveolar lavage fluids of prophylactically treated animals (*n* = 7 per group) were determined by flowcytometry and ELISA, respectively. *P < 0.05, **P < 0.01.

A prophylactic intranasal pre-treatment with either Bet v 1.0101, a mix of five Bet v 1-homologues (Bet v 1.0101, Aln g 1.0101, Cor a 1.0104, Car b 1.0109, and Que a 1.0301), the hybrid proteins FPH, FPH4 all in PBS, or PBS alone was applied in mice prior to allergic sensitization and lung challenge (Fig. 6a). After pre-treatment, animals were multi-sensitized with all five allergens, and an aerosol lung challenge with a mix of birch, alder, hazel, hornbeam, and oak pollen extract was performed. Mice that had received active treatment with either Bet v 1.0101, FPH, or FPH4 showed significantly reduced serum IgE to the WT allergens compared with mice pre-treated with PBS (Fig. 6b). Interestingly, animals protected with the allergen mix did not show a reduced IgE response. Animals actively treated with either the allergen mix, FPH, or FPH4 displayed a significantly reduced lung inflammation after pollen aerosol challenge; however, lung inflammation was most strikingly suppressed by FPH4 pre-treatment as determined by significantly reduced lung eosinophilia (Fig. 7a) and significantly suppressed IL-5 levels in BAL fluids (Fig. 7b). To validate our findings from the prophylaxis model, the experiment was repeated. After immunization with the allergen mix, the IgE response of actively vs. sham pre-treated animals was compared by mediator release assays. Except for Bet v 1.0101, we found that the levels of functional IgE were suppressed by prophylactic treatment. Pre-treatment with the allergen mix and FPH4 most effectively inhibited the IgE production during sensitization, followed by treatment with FPH. Prophylactic treatment with Bet v 1.0101 failed to protect mice from producing specific IgE during immunization (Fig. 6c). Analyses of splenocytes re-stimulated with either parental allergen revealed that prophylactic treatment had a general suppressive effect on the levels of the TH2 cytokines IL-5 and IL-13 compared to pre-treatment with PBS. However, the opposite was observed for IFN-γ (Figure S2). Although not statistically significant due to the small sample number, we found that the cytokine pattern of the pollen allergen mix and FPH4 were very similar showing the highest suppressive or stimulating effects, respectively. Bet v 1 alone represented the least effective pre-treatment option. Analysing BAL fluids of the second prophylaxis model, we found essentially the same picture as for the first trial. Eosinophil levels in the lungs of animals pre-treated with either the allergen mix, FPH, or FPH4 were reduced (Figure S3a), and also IL-5 levels in BAL fluids were suppressed by mix or FPH4 treatment (Figure S3b).

### Fold changes of Bet v 1 translate into increased activation of murine bone marrow-derived dendritic cells

**Figure 8:**
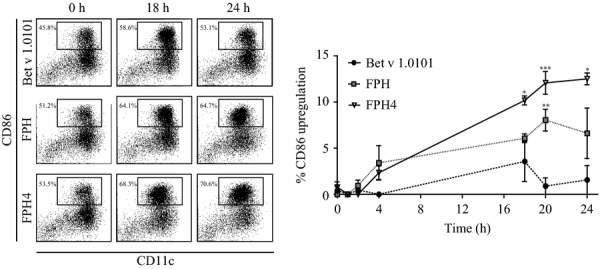
Murine Bone marrow-derived dendritic cells were pulsed with Bet v 1.0101, Fagales pollen hybrid (FPH), or FPH4, respectively, stained for CD11c and CD86, and gated for CD11c expression. Representative FACS profiles are shown; the percentage of CD86 high cells is indicated. Time-dependent CD86 up-regulation is presented as line chart, each point represents the means of three replicates ± CI, and the values are corrected for pre-activated cells. *P < 0.05, **P < 0.01, ***P < 0.001.

Antigen uptake, processing and the subsequent activation of APCs are crucial parameters determining the immunological fate of protein antigens. Thus, murine BMDCs were pulsed with either Bet v 1.0101 or the hybrid proteins, and BMDC activation was analysed by flow cytometry (Fig. 8). Stimulation of BMDCs with Bet v 1.0101 led to weak activation of APCs as indicated by very low up-regulation of CD86 (3.6% compared to un-stimulated cells after 18 h of antigen contact). Of note, this faint activation declined rapidly, and after 24 h of antigen contact, only 1.7% cells were CD86 positive compared with controls. Fagales pollen hybrid activated 5.9% (CD86) of BMDCs after 18 h, whereas FPH4 lead to even stronger up-regulation of 6.6%, respectively. Interestingly, the two hybrid proteins could activate even more cells after 24 h (FPH 10.2% and FPH4 12.5%). Despite an up-regulation of CD86, we could not detect secreted IL-12, TNF-α, or CCL17 (data not shown).

## Discussion

Specific immunotherapy is probably the only treatment capable of altering the natural course of an allergic disease. Although the precise mechanisms of SIT remain a matter of research, the effects of SIT on the pathophysiology as well as the immunological parameters are well described. The treatment shows a very early desensitizing effect on the mast cells as well as basophils combined with a suppressed proliferative activity of allergen-specific T cells paralleled by a reduction of pro-inflammatory cytokines. Moreover, the induction of regulatory T cells secreting IL-10 as well as TGF-β, and during later stages of the treatment, the activation of IgG-producing B cells are crucial parameters determining the clinical success of the intervention 1. Thus, to generate effective allergy therapeutics, it is necessary to tackle both the T cell and the B cell immunity. This selection of the most effective allergen or allergen combination is often difficult especially with closely related allergen families. Bet v 1-like Fagales pollen allergens are such an example. The proteins are highly homologous; several pollen allergens of the family were shown to have the potential to initiate allergic sensitization, and broad IgE as well as T cell cross-reactivity has been reported 6,13,14. Especially in geographical areas where birch is not endemic, other Fagales species initiate allergic reactions. Thus, we thought to create a hybrid allergen by combining known cross-reactive T cell epitopes of the five clinically most relevant Bet v 1-like pollen allergens from birch, hazel, alder, hornbeam, and oak. The Fagales pollen hybrid FPH having the overall length of Bet v 1 showed secondary structure elements similar to Bet v 1-like allergens, and also the hydrodynamic radius of Bet v 1 was preserved. In addition, a fold variant of FPH termed FPH4 was designed by *in silico* mutagenesis. The structural modification led to altered secondary structure elements, accompanied by an increased hydrodynamic radius. As the molecules represent vaccine candidates, human serum IgE binding was considered a crucial parameter concerning safe application in SIT. To investigate the IgE reactivity of the hybrid proteins, ELISAs have been performed using sera of Fagales sensitized patients from two distinct geographical areas, namely Austria, where birch can be considered the most dominant Fagales species, and an area around Rome, Italy, which is described as essentially birch-free, whereas other Fagales, that is, hazel, hornbeam, or oak are endemic 6. For either population, both variants showed reduced IgE-binding capacities; however, this effect was most striking for the fold variant FPH4. The results were confirmed in inhibition experiments, and we found a reduction in the biological activity of both hybrids as determined by mediator release assays of RBL cells passively sensitized with patients′ serum IgE. Again, FPH4 showed the least activity compared with FPH or the parental wild-type allergens. Nevertheless, the hybrids should be able to induce cross-reactive blocking IgG antibodies reactive with WT Fagales allergens. In a mouse model, immunization with the hybrid proteins induced IgG antibodies reactive with all five WT allergens, whereas antibody titres obtained by FPH4 exceeded the titres induced by FPH several fold. Of note, immunization with Bet v 1.0101 induced only low levels of cross-reactive IgG antibodies; however, IgE antibodies induced by Bet v 1.0101 were highly cross-reactive with any WT allergen. Although immunization with FPH or FPH4, respectively, induced cross-reactive IgE, antibody levels were generally lower compared with Bet v 1.0101, and when calculating the IgE:IgG ratio much in favour of potentially protective IgG. It seems that the structural modification of FPH4 boosted the immunogenicity of the protein; however, enough structural elements of Fagales allergens were conserved to induce a potent cross-reactive IgG response. In addition, the fold alteration led to a skewing of the T cell response towards TH1. This is reflected in significantly reduced IL-4 but significantly increased IFN-γ-producing T cells induced by FPH or FPH4 immunization when compared to WT Bet v 1. To investigate the T cell activating properties of both hybrids in detail, mice were immunized with a mix of five parental Fagales allergens and T cells were restimulated with *in vitro* pulsed DCs. With this experiment, we wanted to mimic a situation of multi-sensitization followed by T cells reactivation with either wild-type allergens alone or mixed, or the hybrid proteins, which could be a possible scenario in allergy treatment. Thereby, T cell restimulation with the mix was most effective, followed by restimulation with FPH and FPH4, which was significantly better than restimulation with Bet v 1.0101. This proved that the combination of multiple T cell epitopes of different Fagales allergens is an effective strategy to target T cells specific for different Bet v 1-like proteins. Nevertheless, a murine model system is restricted to one MHC haplotype; thus, T cell epitopes might not be effectively displayed introducing a bias of the T cell response. Based on our findings, it will be very interesting to investigate the T cell activating properties of both hybrid proteins using human-based models, preferably including donors with different geographical/Fagales exposure background. To further characterize the hybrid proteins, we established a prophylactic mouse model and investigated the potential of the molecules to prevent allergic inflammation. Animals received protective treatment, followed by allergic sensitization with a mix of five parental allergens, and lung challenge with an extract mix from birch, hazel, alder, hornbeam and oak pollen. Pre-treatment with the hybrids resulted in significantly reduced lung inflammation reflected by low eosinophil counts and IL-5 levels in BAL fluids. Pre-treatment with Bet v 1.0101 or a mix of the five parental allergens used as control was also successful; however, FPH4 treatment prevented allergic lung inflammation most effectively. At the T cell level, we found that active allergy prophylaxis compared to sham treatment in general suppressed TH2 cytokines, whereas TH1 cytokine levels were boosted. Moreover, we found a reduction of specific IgE induced by allergy prophylaxis with the allergen mix, FPH, and FPH4. In summary, all animal experiments denoted that the allergen mix and FPH4 were most effective in allergy prevention. Moreover, the results were verified in two independent studies. Considering the increased safety profile of FPH4 further underlines its clinical potential as vaccine candidate. Using murine BMDCs, we investigated antigen uptake and presentation, a process that is intimately connected to immunogenicity of protein antigens. As previously reported, we found that Bet v 1.0101 is processed ineffectively by BMDCs 8,18. However, both hybrids induced strong and persistent activation of APCs. Thus, the structure of Bet v 1.0101 seems to harbour unique properties, which prevent efficient antigen uptake by DCs. Obviously, this feature has not been transferred to the hybrid proteins. Moreover, dramatic structural remodelling as induced in FPH4 seems to be highly beneficial for antigen processing. Therefore, we conclude that the interplay between antigen and APC is a crucial mechanism determining the immunological fate of proteins. Especially with FPH4, we can tackle the problem Fagales pollen multi-sensitization, which encourages clinical testing.

## Supporting Information

Figure S1 Inhibition ELISA experiments were performed with sera from Austrian Fagales allergic individuals. Sera were pre-incubated with either the same antigen which was used for coating (black columns), FPH (grey columns), or FPH4 (white columns), respectively.

Figure S2 Splenocytes from mice (*n* = 5 per group) prophylactically treated with the respective antigens or sham were re-stimulated with either parental allergen. Cytokine profiles in supernatants were quantified by FlowCytomix assays.

Figure S3 (a) Eosinophil counts and (b). IL-5 of BAL fluids of prophylactically treated animals (*n* = 5 per group) were determined by flowcytometry and ELISA, respectively.

Table S1 Total IgE measured by EIA (enzyme immunoassay) is presented as IU/L, and specific IgE antibodies determined by ImmunoCAP assays (Thermo Fisher) are presented as kUa/L. Skin prick tests (SPTs) were performed with commercially available extracts and evaluated semi-quantitatively (+ – ≥ 3–<4 mm, ++ – ≥ 4–< 5 mm, +++ – ≥ 5–< 6 mm, ++++ – ≥ 6 mm). F, female; M, male; nd, not determined.
