# Supplementary material for: Allergen hybrids – next generation vaccines for Fagales pollen immunotherapy
Source: Clin Exp Allergy. 2014 Feb 22;44(3):438–49. doi: 10.1111/cea.12250 (PMC4041320; doi:10.1111/cea.12250)
Supplement: Table S1 — Total IgE measured by EIA (enzyme immunoassay) is presented as IU/L, and specific IgE antibodies determined by ImmunoCAP assays (Thermo Fisher) are presented as kUa/L. Skin prick tests (SPTs) were performed with commercially available extracts and evaluated semi-quantitatively (+ – ≥ 3–<4 mm, ++ – ≥ 4–< 5 mm, +++ – ≥ 5–< 6 mm, ++++ – ≥ 6 mm). F, female; M, male; nd, not determined. [file cea0044-0438-sd4.docx]

| **Patient code** | **Country** | **Gender** | **Age** | **Total IgE** | **sIgE birch** | **SPT birch** | **sIgE hazel** | **SPT hazel** | **sIgE oak** | **SPT oak** |
| --- | --- | --- | --- | --- | --- | --- | --- | --- | --- | --- |
| 646036 | Austria | F | 27 | 740 | 62.1 | nd | nd | nd | nd | nd |
| 646077 | Austria | F | 17 | 1591 | 59.0 | nd | nd | nd | nd | nd |
| 646308 | Austria | F | 31 | 72.3 | 50.4 | nd | nd | nd | nd | nd |
| 646389 | Austria | F | 33 | 211 | >100 | nd | nd | nd | nd | nd |
| 646440 | Austria | M | 39 | 289 | 78.5 | nd | nd | nd | nd | nd |
| 646824 | Austria | F | 18 | 148 | 72.3 | nd | nd | nd | nd | nd |
| 646859 | Austria | F | 18 | 93.9 | 51.6 | nd | nd | nd | nd | nd |
| 646880 | Austria | M | 13 | 277 | 66.6 | nd | nd | nd | nd | nd |
| 646898 | Austria | M | 10 | 114 | 64.1 | nd | nd | nd | nd | nd |
| 646942 | Austria | F | 33 | 201 | 73.3 | nd | nd | nd | nd | nd |
| 646951 | Austria | M | 16 | 705 | >100 | nd | nd | nd | nd | nd |
| 646966 | Austria | F | 49 | 133 | 93.6 | nd | nd | nd | nd | nd |
| 647064 | Austria | M | 65 | 998 | 56.4 | nd | nd | nd | nd | nd |
| 647095 | Austria | M | 69 | 344 | >100 | nd | nd | nd | nd | nd |
| 647099 | Austria | F | 27 | 598 | >100 | nd | nd | nd | nd | nd |
| 647122 | Austria | F | 16 | 1163 | >100 | nd | nd | nd | nd | nd |
| 3813-CR | Italy | M | 11 | nd | 11,9 | ++++ | 56 | ++++ | 24 | +++ |
| 3789-FE | Italy | F | 30 | nd | 6,3 | ++++ | 4,6 | ++++ | 3,6 | ++++ |
| 3763-LP | Italy | M | 13 | nd | 1,5 | ++ | 1,3 | ++++ |  | + |
| 3735-NV | Italy | F | 16 | nd | 49,2 | ++++ | 79,7 | ++++ | 30,3 | ++ |
| 3580-TG | Italy | M | 12 | nd | 3,6 | +++ | 7,8 | +++ | 11,3 | +++ |
| 3574-CG | Italy | M | 9 | nd | 17,6 | ++++ | 22,4 | ++++ | 5,3 | +++ |
| 3553-MV | Italy | F | 13 | nd | 0,9 | ++++ | 6,1 | ++++ | 0,3 | ++ |
| 3497-CM | Italy | M | 11 | nd | 10,2 | +++ | 7 | +++ | 9,5 | +++ |
| 3490-RF | Italy | M | 20 | nd | 7,5 | +++ | 3,6 | ++++ | 2,2 | ++++ |
| 3482-RP | Italy | F | 39 | nd | 16,6 | ++++ | 18,3 | ++++ |  | +++ |
| 3440-PM | Italy | M | 42 | nd | 9,1 | ++++ | 7,5 | ++++ | 10,3 | ++++ |
| 3434-FP | Italy | M | 37 | nd | 16,7 | ++++ | 16,8 | ++++ | 10,1 | ++++ |
| 3201-SV | Italy | F | 12 | nd | 4,5 | ++++ | 14,6 | ++++ |  | ++++ |
| 3169-PV | Italy | F | 71 | nd | 4,7 | ++++ | 7,1 | +++ | 1 | +++ |
| 3155-BA | Italy | F | 31 | nd | 13,8 | +++ | 6,3 | +++ |  | ++++ |
| 3082-PL | Italy | M | 10 | nd | 29,2 | ++++ | 52,9 | ++++ |  | ++ |
| 2949-DMS | Italy | F | 19 | nd | 1 | +++ | 2,5 | ++++ | 0,1 | - |
| 2900-CM | Italy | F | 36 | nd | 4,6 | ++++ | 2,5 | ++++ | 0,1 | ++++ |
